# Histopathological Lung Findings in COVID-19 B.1.617.2 SARS-CoV-2 Delta Variant

**DOI:** 10.3390/jpm13020279

**Published:** 2023-01-31

**Authors:** Ionuț Isaia Jeican, Patricia Inișca, Dan Gheban, Vlad Anton, Mihaela Lazăr, Mihaela Laura Vică, Daniela Mironescu, Codrin Rebeleanu, Carmen Bianca Crivii, Maria Aluaș, Silviu Albu, Costel Vasile Siserman

**Affiliations:** 1 Department of Anatomy and Embryology, Iuliu Hatieganu University of Medicine and Pharmacy, 400006 Cluj-Napoca, Romania; 2 Department of Pathology, County Emergency Hospital Deva, 330084 Deva, Romania; 3 Department of Pathology, Iuliu Hatieganu University of Medicine and Pharmacy, 400006 Cluj-Napoca, Romania; 4 Department of Pathology, Emergency Clinical Hospital for Children, 400370 Cluj-Napoca, Romania; 5 Department of Medical Biochemistry, Iuliu Hatieganu University of Medicine and Pharmacy, 400349 Cluj-Napoca, Romania; 6 Viral Respiratory Infections Laboratory, Cantacuzino National Military-Medical Institute for Research and Development, 050096 Bucharest, Romania; 7 Institute of Legal Medicine, 400006 Cluj-Napoca, Romania; 8 Department of Cell and Molecular Biology, Iuliu Hatieganu University of Medicine and Pharmacy, 400349 Cluj-Napoca, Romania; 9 Department of Legal Medicine, Iuliu Hatieganu University of Medicine and Pharmacy, 400006 Cluj-Napoca, Romania; 10 Department of Oral Health, Iuliu Hatieganu University of Medicine and Pharmacy, Victor Babeș Str., No. 15, 400012 Cluj-Napoca, Romania; 11 Department of Head and Neck Surgery and Otorhinolaryngology, University Clinical Hospital of Railway Company, Iuliu Hatieganu University of Medicine and Pharmacy, 400015 Cluj-Napoca, Romania

**Keywords:** COVID-19, B.1.617.2, Delta variant, histopathology, lung

## Abstract

Background: The Delta variant (Pango lineage B.1.617.2) is one of the most significant and aggressive variants of SARS-CoV-2. To the best of our knowledge, this is the first paper specifically studying pulmonary morphopathology in COVID-19 caused by the B.1.617.2 Delta variant. Methods: The study included 10 deceased patients (40-83 years) with the COVID-19 Delta variant. The necrotic lung fragments were obtained either by biopsy (six cases) or autopsy (four cases). Tissue samples were subjected to virology analysis for identification of the SARS-CoV-2 variant, histopathology, and immunohistochemistry (anti-SARS coronavirus mouse anti-virus antibody). Results: Virology analysis identified B.1.617.2 through genetic sequencing in eight cases, and in two cases, specific mutations of B.1.617.2 were identified. Macroscopically, in all autopsied cases, the lung had a particular appearance, purple in color, with increased consistency on palpation and abolished crepitations. Histopathologically, the most frequently observed lesions were acute pulmonary edema (70%) and diffuse alveolar damage at different stages. The immunohistochemical examination was positive for proteins of SARS-CoV-2 in 60% of cases on alveolocytes and in endothelial cells. Conclusions: The histopathological lung findings in the B.1.617.2 Delta variant are similar to those previously described in COVID-19. Spike protein-binding antibodies were identified immunohistochemically both on alveolocytes and in the endothelial cells, showing the potential of indirect damage from thrombosis.

## 1. Introduction

Since the World Health Organization (WHO) classified the coronavirus disease 2019 (COVID-19) as a pandemic in March 2020, among the multiple variants of the virus causing it, severe acute respiratory syndrome coronavirus 2 (SARS-CoV-2) has arisen as a result of viral evolution [1]. Based on the epidemiological data from the WHO, in occidental countries, the predominant variants of SARS-CoV-2 were the variant of concern Alpha (B.1.1.7), first described in the United Kingdom in September 2020, followed by Delta (B.1.617.2), first reported in India in October 2020, and then Omicron (B.1.1.529), first reported in South Africa in November 2021 [2,3].

One of the most significant was the Delta variant (Pango lineage B.1.617.2), first designated by the WHO as a variant of concern (VOC) in May 2021 [3]. The Delta variant had already outpaced the other abovementioned variants all over the world by late spring 2021 [4]. The Delta variant was shown to be 1.4 to 1.64 times more transmissible than the Alpha variant [5,6]. In addition, a series of studies have shown that the Delta variant was more aggressive than the previous strains [7,8].

The literature data suggest that, in addition to human-to-human contact, SARS-CoV-2 may spread via fecal–oral and aerosols [9]. The underlying mechanism of COVID-19 pneumonia is primarily altered lung perfusion (lung edema, atelectasis, and, therefore, recruitability) [10]. Almost all studies describe COVID-19 lung as being heavy, firm, and edematous. The most reported finding in COVID-19 is diffuse alveolar damage (DAD) in the lung histology, hyaline membranes, type II pneumocytes hyperplasia, the presence of lymphocytes, macrophages, and multinucleated giant cells [11]. COVID-19 can cause venous and arterial thrombotic complications through a combination of thrombocytopenia, prolonged prothrombin time, and elevated D-dimer, likely due to disseminated intravascular coagulation or thrombotic microangiopathy [12]. In addition, it seems that secretory phospholipase 2 (a mediator of the inflammatory cascade) in high levels can be correlated with COVID-19 severity [13,14].

The aim of the study was to observe the pulmonary histopathological characteristics of COVID-19 infection caused by the B.1.617.2 Delta variant in a batch of deceased patients.

## 2. Materials and Methods

The samples were harvested from deceased patients from the Department of Pathology, County Emergency Hospital Deva and Institute of Legal Medicine Cluj-Napoca, while complying with Romanian Law and specific international and national recommendations for COVID-19 [1,2,3,4,5,6,7,8,9,10,11,12,13,14,15,16,17], during December 2021–February 2022. 

The harvesting protocol for this study was approved by the County Emergency Hospital Deva Ethics Committee, the Administrative Direction of the County Emergency Hospital Deva under No. 32692/2021, and the Administrative Department of the Institute of Legal Medicine Cluj-Napoca under No. 4353/XII/614/2021.

The study group consisted of 10 patients with a diagnosis of COVID-19 infection (B.1.617.2 SARS-CoV-2 Delta variant) confirmed postmortem from lung tissue.

### 2.1. Sampling

Two necrotic lung fragments were obtained 12 h after death, either by biopsy (approximative 1 cm incision made under the 4th rib on the right middle axillary line, then 2 pulmonary samples 5/5 mm were harvested with forceps) or by autopsy (the opening of the thoraco–pulmonary cavity, the complete extraction of lungs, and the collection of 2 pulmonary samples of 5/5 mm from the regions of maximum condensation) (Table 1): one for SARS-CoV-2 virology analyses (stored in a viral transport medium BioSci virus sampling tube model FBY, Darkewe Biotech Co. Ltd., Shenzhen, China; the tubes were stored immediately after collection in a freezer at a temperature of −20 °C, then they were transferred to a freezer at a temperature of −80 °C over the next 24 h and stored until analysis), and one for histopathology (stored in 7% formaldehyde).

### 2.2. Virology Analysis for SARS-CoV-2 Variant

Total RNA isolation was performed using NucleoSpin RNA for tissue (Macherey-Nagel, Dueren, Germany) and MagnaPure 96 DNA and Viral NA (Roche Diagnostics, Indianapolis, Indiana, USA) from viral transport medium, according to the manufacturer’s instructions. The Allplex 2019-nCoV assay (Seegene, Seoul, South Korea) was designed for amplifying three viral targets: the E gene (specific of the subgenus Sarbecovirus), the N, and the RdRP genes (both specifics of SARS-CoV-2), using a QuantStudio 7 Pro Real-Time PCR System (Applied Biosystems) and/or a Bio-Rad CFX96 instrument (Bio-Rad, Hercules, CA, USA). 

The Allplex SARS-CoV-2 Variants I and II RT-PCR assay (Seegene, Seoul, South Korea) was used to detect mutations of different variants (N501Y, E484K, delHV69/70, L452R, W152C, K417N, and K417T) as a first line screening tool according to manufacturer’s instructions.

All SARS-CoV-2 RNA-positive samples underwent real-time whole-genome sequencing. RNA preparation and amplification were performed following protocols published by the ARTIC network using the V3 version of the ARTIC primer set from Integrated DNA Technologies (Coralville, IA, USA) to create tiled amplicons across the SARS-CoV-2 genome. Libraries were prepared using Nextera DNA Flex library preparation kit and MiSeq reagent cartridge V2 (Illumina, San Diego, CA, USA) [18]. All samples were examined by the same experienced investigator (M.L.).

### 2.3. Histopathology

The samples were fixed in 7% formaldehyde for 5 days, after which the samples were oriented and placed in cassettes. Tissue processing was performed using a vacuum infiltration processor, Tissue-Tek VIP 5 Jr (Sakura, Alphen aan den Rijn, Netherlands). Paraffin embedding and sectioning were performed using the Tissue-Tek TEC 6 system (Sakura, Alphen aan den Rijn, Netherlands) and Accu-Cut SRM 200 Rotary Microtome (Sakura, Alphen aan den Rijn, Netherlands). Slide staining was performed using the automated slide stainer Tissue-Tek Prisma Plus (Sakura, Alphen aan den Rijn, Netherlands), according to the internal staining protocol, using Mayer Modified Hematoxylin (Titolchimica, Rovigo, Italy) and Eosine solution (10 g Eosine B in 1000 mL distilled water). 

Immunohistochemistry was performed automatically on 3 μm thick sections of formalin-fixed and paraffin-embedded tissues with MD Stainer (Vitro Master Diagnostica^®^, Granada, Spain) using ethylenediaminetetraacetic acid (EDTA), at pH = 9, for antigen retrieval. We used anti-SARS coronavirus NP mouse anti-virus antibody (clone B46F, Invitrogen, Waltham, MA, USA) at a 1:100 dilution for the immunohistochemical assessment. Positive cells are colored in brown.

Microscopic examination was performed by the same experienced pathologist (D.G.), using a Leica DM1000 clinical microscope (Leica, Wetzlar, Germany) with a dedicated image acquisition camera and software. All sections were examined by the same experienced investigator (D.G.).

## 3. Results

We analyzed 10 patients (6 men and 4 women), aged between 40 and 83 years (mean age 66.4, σ = 11.41), who died from a severe form of COVID-19 during the Delta wave in Romania (December 2021–February 2022). Out of the patients included in the study, seven came from the Department of Pathology, County Emergency Hospital Deva, and three came from the Institute of Legal Medicine Cluj-Napoca. Out of the 10 patients, a complete autopsy was performed for 4 patients, and a postmortem lung tissue biopsy was performed for 6 patients (Table 1).

Virology analysis for the SARS-CoV-2 variant from lung necrotic samples identified B.1.617.2 through genetic sequencing in eight cases, and in two cases, specific mutations of B.1.617.2 were identified (Table 1).

**Figure 1:**
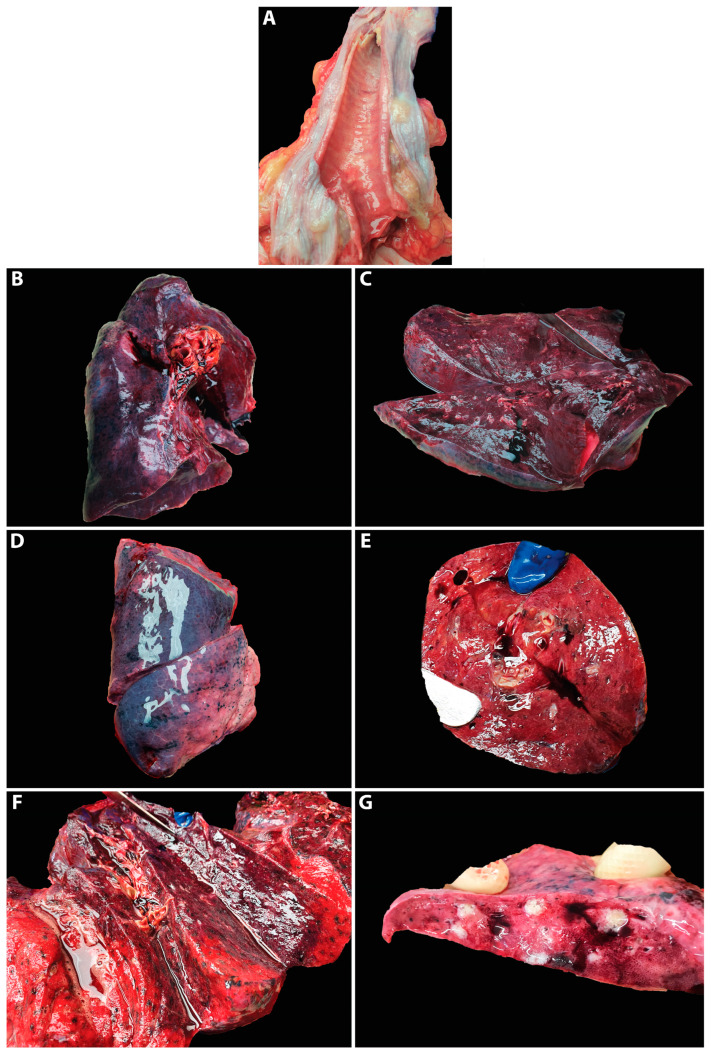
Macroscopic aspects of lung infected with B.1.617.2 SARS-CoV-2 Delta variant: (**A**) acute pulmonary edema; foamy liquid in trachea; (**B**–**F**) condensed, violaceous lung; (**G**) expression of the pulmonary section in bronchopneumonia.

At the macroscopic examination, acute pulmonary edema was identified in 70% of the autopsied cases (*n* = 7/10) (Figure 1A). In all autopsied cases, the lung had a particular appearance, purple in color, with increased consistency on palpation and abolished crepitations (Figure 1B–F). One of the autopsied cases presented bronchopneumonia, and purulent and hemorrhagic secretions were revealed when the lung section was expressed (Figure 1G).

**Figure 2:**
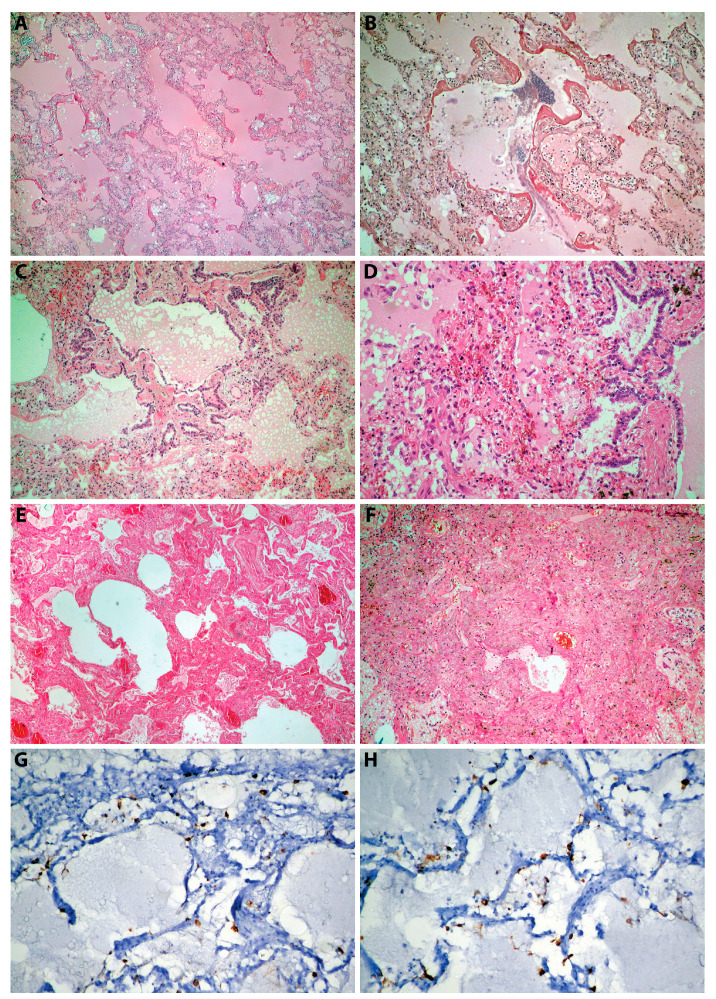

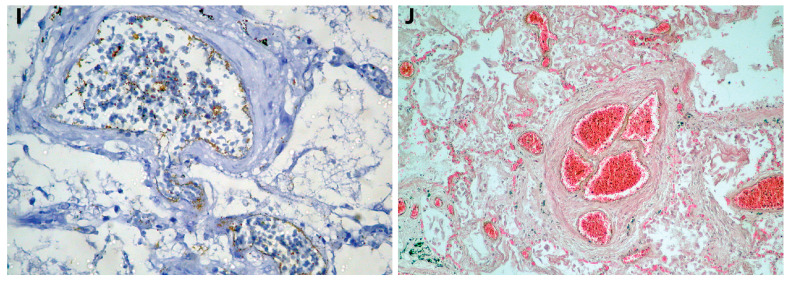
Histopathology and immunohistochemistry of lung infected with B.1.617.2 SARS-CoV-2 Delta variant: (**A**) massive acute pulmonary edema (case No. 1); (**B**) DAD I (case No. 2); (**C**) acute pulmonary edema, DAD 2 (case No. 4); (**D**) DAD 2 (detail); (**E**) DAD 3, interstitial fibrosis (case No. 3); (**F**) DAD 3 (case No. 10); (**G**,**H**) positive proteins of SARS-CoV-2 in alveolocytes (case No. 2); (**I**) positive proteins of SARS-CoV-2 in endothelial cells (case No. 4); (**J**) plexogenic lesion (case No. 4).

At the histopathological examination, the most frequently observed lesions were acute pulmonary edema (70%, *n* = 7/10) (Figure 2A), DAD 1 (50%, *n* = 5/10) (Figure 2B), DAD 2 (40%, *n* = 4/10) (Figure 2C–D), and DAD 3 (20%, *n* = 2/10) (Figure 2E–F).

The immunohistochemical examination was positive for proteins of SARS-CoV-2 in 60% of cases (*n* = 6/10): five cases are described as positive using immunohistochemistry to proteins of SARS-CoV-2 on alveolocytes (Figure 2G–H), one in an endothelial cell (Figure 2I), and four others were negative.

## 4. Discussion

The B.1.617.2 SARS-CoV-2 Delta variant has particularly attracted attention due to its aggressiveness. B.1.617.2 is one of the most aggressive strains of the SARS-CoV-2 virus to appear so far. A study on Singapore patients revealed that patients infected with the Delta variant had an adjusted odds ratio of 4,9 for oxygen requirement, admission to an intensive care unit (ICU), or death, compared to patients infected with other VOCs [7]. A Canadian study showed similar, albeit smaller increases in disease severity caused by the Delta variant compared to the wild-type (non-VOC) virus: a 108% higher risk of hospitalization, 234% higher risk of ICU admission, and 132% higher risk of death [8]. The Delta variant was shown to be resistant to the therapeutic monoclonal antibody bamlanivimab while remaining susceptible to etesivimab, casirivimab, and imdevimab [19]. This, and another study, assessed the Delta variant to be four to six times more resistant to neutralization by convalescent serum and three to eight times more resistant to serum from vaccinated individuals [20]. However, a systematic review and meta-analysis of 15 studies showed that a full vaccination schedule with either the AstraZeneca, Pfizer, or Moderna vaccines had an effectiveness of more than 77,5% in preventing infection, more than 88% in preventing hospitalization, and more than 91% in preventing death [21]. Another systematic review and meta-analysis comparing vaccine effectiveness (VE) against the Delta variant to VE against the Alpha variant showed a VE reduction of 10-20% for non-severe outcomes but no difference in VE for hospitalization between the Delta and Alpha strains [22].

Our results did not reveal special pulmonary histopathological aspects for the B.1.617.2 SARS-CoV-2 Delta variant compared to those known so far in COVID-19. SARS-CoV-2 causes DAD, which can lead to acute respiratory distress syndrome (ARDS) in some patients [23]. Macroscopically, the lungs of COVID-19 patients increase in weight, their consistency becomes firmer, and they become reddish–brown from lung hepatization [24]. COVID-19 DAD follows the general histopathological stages of DAD, from exudative to organizing and finally fibrotic [25]. 

The exudative stage is characterized by the formation of a hyaline membrane along the alveolar wall alongside immune cell infiltration of the alveolar wall. In the organizing stage, more immune cells, such as macrophages and plasma cells, infiltrate through the alveolar wall, and squamous cell metaplasia and type II alveolar epithelial cell hyperplasia can be observed. In the fibrotic stage, proliferating fibroblasts in the interstitium and collagen fiber deposits obstruct the alveolar space and modify the alveolar structure. Autopsies on the lungs of deceased patients revealed that the affected lungs could manifest all three of the aforementioned DAD stages at once, suggesting a gradual spread and progression of the infection throughout the lung, ultimately leading to respiratory failure [25]. It should be noted that COVID-19-related DAD is morphologically indistinguishable from DAD due to other causes [26].

The lungs may also be affected by edema, hemorrhage, loss of cellular integrity, various vascular abnormalities, and, in some cases, lobe infarction due to thrombosis of large pulmonary vessels [27]. Most anatomical and histopathological characterizations of COVID-19 lungs pre-date the identification of the Delta variant as a VOC, but no studies have been found to date suggesting specifically that the Delta variant would have different effects on the anatomical and histopathological levels.

A detailed pathophysiological mechanism of the induction of acute lung injury (including DAD) by SARS-CoV-2 has been proposed in a 2021 review on the pulmonary pathology of COVID-19 [28]. Single-cell transcriptomic data revealed that, in the respiratory system, ACE2 is expressed primarily in type II alveolar cells in the lungs and in goblet cells within the nasal mucosa and the bronchi [29]. SARS-CoV-2 viral particles in the respiratory system have been found along the cell membranes of type II alveolar cells but also in endothelial cells, although this latter localization is still a matter of debate [28]. In their work, the authors observed spike protein-binding antibodies on the alveolocytes of five subjects and in the endothelial cells of one subject. Thus, we can consider not only the mechanisms of direct lung damage with infection but especially the indirect damage from thrombosis.

Case No. 4 from our study presented with a plexiform lesion (Figure 2J), a hallmark of severe pulmonary hypertension [30]. ARDS can be frequently complicated by pulmonary hypertension. Physiopathologically, pulmonary hypertension is caused by vascular obstructions, pulmonary vasoconstriction, and microthrombosis. Subsequently, in the subacute and chronic phases of ARDS, the vascular remodeling and proliferation of smooth muscle cells contribute to the development of pulmonary hypertension [31,32,33]. The prevalence of pulmonary hypertension in patients with COVID-19 is 12% [34]. In addition, the studies show that pulmonary hypertension may be a long-term sequela of COVID-19, which may be a major public health issue in the future [35]. Although we do not have the antemortem clinical data in the case No. 4, we consider that the plexiform lesion is not correlated directly with COVID-19: most studies show that it takes a few weeks until the plexiform lesions appear after the establishment of severe pulmonary hypertension [36,37].

Some limitations must be highlighted related to our study. The first is the reduced number of patients included in the study, since between December 2021–February 2022, it was the total number of patients which filled the conditions of inclusion (diagnosis of the COVID-19 infection B.1.617.2 SARS-CoV-2 Delta variant, confirmed postmortem from lung tissue). Second, for two patients, we did not obtain the virus genetic sequencing (this can be explained by low viral loads and/or poor nucleic acid quality). Third, at the time of the immunohistochemical exam, we were limited only to the identification of the spike protein, and we could not test for other SARS-CoV-2 proteins.

## 5. Conclusions

Although the B.1.617.2 Delta variant is one of the most aggressive strains of the SARS-CoV-2 virus, the histopathological lung findings are similar to those previously described in COVID-19. Spike protein-binding antibodies were identified immunohistochemically both on alveolocytes and in the endothelial cells, showing the potential of indirect damage from thrombosis.

## Figures and Tables

**Table 1:** Research design and molecular, histopathology, and immunohistochemistry results.

No. of Case, Sex, Age	Medical Institution of the Patients	Sampling of Lung Tissue	SARS-CoV-2 Variant/ GISAID	Histopathology Result	Immunohistochemistry Result
1, M, 61	Department of Pathology, County Emergency Hospital Deva	Postmortem biopsy	B.1.617.2/ EPI_ISL_5241180	Acute pulmonary edema (Figure 2A), DAD 1	Negative
2, M, 66	Department of Pathology, County Emergency Hospital Deva	Postmortem biopsy	B.1.617.2/ EPI_ISL_5858608	Acute pulmonary edema, DAD 1 (Figure 2B)	Positive proteins of SARS-CoV-2 in alveolocytes (Figure 2G–H)
3, M, 83	Department of Pathology, County Emergency Hospital Deva	Postmortem biopsy	B.1.617.2/ EPI_ISL_5858607	DAD 3 (Figure 2E)	Positive proteins of SARS-CoV-2 in alveolocytes
4, F, 69	Department of Pathology, County Emergency Hospital Deva	Autopsy (Figure 1D–E)	B.1.617.2/ Positive for screening (L452R)	Acute pulmonary edema, DAD 1, focal DAD 2 (Figure 2C–D), multifocal organizing pneumonia, pulmonary microthrombosis, plexogenic lesion (Figure 2J)	Positive proteins of SARS-CoV-2 in endothelial cells (endothelithis COVID-19) (Figure 2I)
5, M, 63	Department of Pathology, County Emergency Hospital Deva	Postmortem biopsy	B.1.617.2/ EPI_ISL_15764977	Acute pulmonary edema, DAD 1, focal DAD 2	Positive proteins of SARS-CoV-2 in alveolocytes
6, F, 70	Department of Pathology, County Emergency Hospital Deva	Postmortem biopsy	B.1.617.2/EPI_ISL_15764981	Acute pulmonary edema, DAD 1	Negative
7, F, 64	Department of Pathology, County Emergency Hospital Deva	Postmortem biopsy	B.1.617.2/Positive for screening (L452R)	DAD 2	Positive proteins of SARS-CoV-2 in alveolocytes
8, F, 71	Institute of Legal Medicine Cluj-Napoca	Autopsy (Figure 1A–C)	B.1.617.2/ EPI_ISL_15764979	Acute pulmonary edema, areas of alveolar collapse, DAD 2	Negative
9, M, 77	Institute of Legal Medicine Cluj-Napoca	Autopsy (Figure 1G)	B.1.617.2/ EPI_ISL_15764978	Bronchopneumonia, alveolar proteinosis	Negative
10, M, 40	Institute of Legal Medicine Cluj-Napoca	Autopsy	B.1.617.2/EPI_ISL_15764980	Acute pulmonary edema, DAD 3 (Figure 2F), alveolar collapse	Positive proteins of SARS-CoV-2 in alveolocytes

## Data Availability

The autopsy results are available at the Department of Pathology, County Emergency Hospital Deva and the Institute of Legal Medicine in Cluj-Napoca Romania; contact: patricia.bilei@gmail.com; cvsiserman@gmail.com. The histopathology and immunohistochemistry results are available at the Department of Anatomy and Embryology, Iuliu Hatieganu University of Medicine and Pharmacy, Cluj-Napoca, Romania; contact: jeican.ionut@umfcluj.ro. The virology analysis results are available at the Viral Respiratory Infections Laboratory, Cantacuzino National Military-Medical Institute for Research and Development, Bucharest, Romania; contact: lazar.mihaela@cantacuzino.ro.
